# Ideal body weight-based determination of minimum oral calories beneficial to function and survival in ALS

**DOI:** 10.3389/fneur.2023.1286153

**Published:** 2023-11-08

**Authors:** Ryutaro Nakamura, Mika Kurihara, Shuhei Kobashi, Yoshitaka Tamaki, Nobuhiro Ogawa, Akihiro Kitamura, Isamu Yamakawa, Shigeki Bamba, Tomoya Terashima, Makoto Urushitani

**Affiliations:** ^1^Department of Neurology, Shiga University of Medical Science, Otsu, Japan; ^2^Division of Clinical Nutrition, Shiga University of Medical Science, Otsu, Japan; ^3^Department of Fundamental Nursing, Shiga University of Medical Science, Otsu, Japan

**Keywords:** amyotrophic lateral sclerosis, nutritional therapy, ideal body weight, metabolism, prognosis

## Abstract

**Introduction:**

This study sought to identify the optimal caloric intake to improve function and survival in ALS patients by comparing oral intake per ideal body weight (IBW) and its discrepancy with total energy expenditure (TEE) using the Shimizu formula.

**Methods:**

A retrospective analysis of 104 ALS patients was conducted, categorizing them based on their average intake during the first week after admission using two primary intake cutoffs: 25 kcal/kgIBW and 30 kcal/kgIBW. The variance between oral intake and TEE was also evaluated using −300 kcal and 0 kcal as reference points.

**Results:**

Oral caloric intake per IBW and functional decline rate (rs = −0.35, *p* < 0.001), but the variance from TEE was not significantly correlated (−0.11, *p* = 0.27). Survival data showed that patients consuming less than 25 kcal/kgIBW had a median survival of 24 months, increasing to 38 months for those consuming between 25–30 kcal/kgIBW and 63 months for those consuming 30 kcal/kgIBW or more. Deviations from the TEE did not significantly affect survival (*p* = 0.36). Among patients consuming less than their TEE, those consuming less than 25 kcal/kgIBW had a shorter median survival (24 months) compared to their counterparts (46 months) (*p* = 0.022). Consumption of less than 25 kcal/kgBW emerged as a significant negative predictor of patient outcome, independent of factors such as age, gender or disease progression.

**Discussion:**

Intakes of 25 kcal/kgIBW or more are correlated with improved ALS outcomes, and larger, multi-regional studies are recommended for deeper insights.

## Introduction

Amyotrophic lateral sclerosis (ALS) is a neurodegenerative disease characterized by the paralysis of limb and respiratory muscles due to the loss of upper and lower motor neurons. Without ventilator support, patients usually die within 2–4 years of symptom onset (1). Weight loss is observed in approximately 70% of ALS patients, mainly because of loss of oral intake and metabolic alterations (2). A low body mass index (BMI) at diagnosis is an indicator of poor prognosis (3), and post-diagnosis weight gain is reported to be associated with a longer survival (4). Early initiation of gastrostomy contributes to weight maintenance (5), and high-calorie therapy may delay disease progression, particularly in those with rapid progression (6). In this manner, the accumulated evidence underscores the importance of nutritional management.

For maintaining body weight, it is essential that total energy intake exceeds the total energy expenditure (TEE). Typically, TEE is estimated using the Harris-Benedict equation (7). In ALS patients, resting energy expenditure is elevated (8, 9), suggesting the need for a predictive formula for TEE that accounts for this increase. Kasarskis et al. assessed TEE in ALS patients using a double-labeled water method (10), and Shimizu et al. conducted similar research on Japanese ALS patients, and proposed a predictive formula for TEE, referred to here as the “Shimizu formula” (11). TEE measurements by the Shimizu formula incorporate the Revised Amyotrophic Lateral Sclerosis Functional Rating Scale (ALSFRS-R) and BMI. This assessment may overwhelm the daily intake of ALS patients, especially in the early phase. Indeed, we occasionally encounter situations where patients face challenges in consuming the estimated amount orally. These situations have prompted us to seek additional calorie indicators to ensure that patients who have difficulty following the Shimizu formula have a favorable prognosis.

In the field of metabolism and nutrition, such as diabetes, energy intake recommendations are based on the ideal body weight (IBW), with an intake of approximately 25-35 kcal/kgIBW recommended depending on activity levels (12). The simplicity of this calculation is its strength, and since it’s a consensus metric for caloric administration in other diseases, it is easy for multi-disciplinary teams to share. However, its utility in the nutritional treatment of ALS patients has not been studied.

In this study, we retrospectively analyzed the relationship between survival duration and oral intake, such as energy intake per IBW and difference from TEE, and proposed a new calorie indicator to be used with the Shimizu formula for ALS patients to avoid poor prognosis in survival.

## Materials and methods

### Inclusion criteria and ethics

This single-center retrospective study included patients diagnosed with ALS between March 2016 and July 2023 at Shiga University of Medical Science Hospital, according to either the revised El Escorial criteria (13) being rated as possible or higher, or meeting the Gold Coast criteria (14). The study was approved by the ethics committee of Shiga University of Medical Science Hospital (approval number: R2023-072).

### Characteristics

We employed the data gathered during the initial admission for diagnostic purpose or for treatment with edaravone or percutaneous endoscopic gastrostomy (PEG). Data were extracted from medical records, including age at admission, gender, body weight (B.W.; before illness, at first visit, and during caloric assessment), height, the number of months from onset to first consultation and caloric assessment, presence or absence of dysphagia, El Escorial criteria, PEG using during the disease course, and %vital capacity (V.C.). IBW was calculated as follows:

$IBW=22\times \mathrm{height}\left(m\right)\times \mathrm{height}\left(m\right)$

The rate of weight loss was evaluated using the following weight change rate:

$\begin{array}{l} \Delta BW\;\left(/\mathrm{month}\right)=\left(\mathrm{premorbid}\;\mathrm{weight}:\mathrm{weight}\;\mathrm{at}\;\mathrm{first}\;\mathrm{visit}\right)/\\ \mathrm{number}\;\mathrm{of}\;\mathrm{months}\;\mathrm{from}\;\mathrm{onset} \end{array}$

The rate of disease progression was evaluated using the following decline in ALSFRS-R:

$\begin{array}{l} \mathrm{\Delta ALSFRS}:R=\left(48:\mathrm{ALSFRS}:R\;\mathrm{at}\;\mathrm{diagnosis}\right)/\\ \mathrm{number}\;\mathrm{of}\;\mathrm{months}\;\mathrm{from}\;\mathrm{onset} \end{array}$

### Oral intake assessment

Oral intake data during the initial hospitalization were evaluated. Upon admission, the ALSFRS-R was initially assessed for the majority of patients. However, data was missing for certain individuals, particularly those for whom the probability of ALS was deemed low at the time of admission. The resting metabolic rate (RMR) was then calculated using the Harris-Benedict equation (7), and TEE was derived using the Shimizu formula (11).

For men:

$\begin{array}{l} RMR=66.47+\left[13.75\times \mathrm{weight}\left(\mathrm{kg}\right)\right]+\left[5.00\times \mathrm{height}\left(\mathrm{cm}\right)\right]:\\ \left(6.76\times \mathrm{age}\right) \end{array}$

For women:

$\begin{array}{l} RMR=655.1+\left[9.56\times \mathrm{weight}\left(\mathrm{kg}\right)\right]+\left[1.85\times \mathrm{height}\left(\mathrm{cm}\right)\right]:\\ \left(4.68\times \mathrm{age}\right) \end{array}$

$\mathrm{TEE}\;\left(\mathrm{kcal}\right)=\left(1.67\times RMR\right)+\left(11.8\times \mathrm{ALSFRS}:R\right):680$

The amount of food provided during hospitalization was determined based on the Shimizu formula since the publication. The caloric needs of patients admitted prior to formula adaptation were determined using the Harris-Benedict equation. All the patients had been informed about the importance of food consumption, and food intake was visually evaluated and documented by ward nurses. To ensure a stable intake assessment, the average intake over seven days post-admission was considered the daily oral intake. For patients undergoing gastrostomy within one week of admission, the average intake up to the day before the procedure was used.

### Metabolic assessment

To evaluate the relationship between oral intake and metabolism, we measured the resting energy expenditure (mREE; kcal) using an indirect calorimeter (Aeromonitor^®^ AE310S, Minato Medical Science Co., Osaka, Japan). The examination was conducted for 10 min in the morning after an overnight fast with patients in a supine position. Before starting, participants rested for 30 min (15). The Respiratory Quotient (R.Q. = VCO2/VO2) was calculated, and the mREE was determined using the Weir formula (16). The evaluation of hypermetabolism varies across races (17), and there is no consensus for Japanese patients. In this study, similar to our previous research (18), we used the REE per lean soft tissue mass (LSTM; kg) as the main metabolic indicator. LSTM was measured through a bioelectrical impedance analysis with a body composition analyzer (InBody S10; InBody, Tokyo, Japan), conducted consecutively with mREE measurement. Other metabolic markers included data on body temperature measured in the axilla in the morning during the hospitalization. From the medical records of the first week after admission, we obtained the average body temperature of five days, excluding the highest and lowest values. We also collected data on High-density lipoprotein cholesterol (HDL) (19, 20) and Low-density lipoprotein cholesterol (LDL) (20, 21) from blood tests taken at the first visit, including non-fasting outpatient blood tests because HDL and LDL tend to decrease over time in patients with ALS (20).

### Follow-up

Survival time was the time since the onset to death or tracheotomy invasive ventilation (TIV), with a cutoff point of 31st July 2023. To mitigate the influence of the life-prolonging effect of TIV on survival length, we considered both TIV and death as endpoints. For those who survived past this date, their survival time was censored.

### Statistics

Statistical analysis was performed using EZR version 1.53 (Saitama Medical Center, Jichi Medical University, Tochigi, Japan) (22). Continuous variables were presented as median and quartiles. Comparisons between two groups were made using the Mann–Whitney U test and Fisher’s exact probability test. Correlations were assessed using the Spearman correlation coefficient. The endpoints were defined as death or tracheostomy. Survival duration from onset was evaluated using Kaplan–Meier curves and the Log-rank test. For oral intake per IBW, we referred to the recommended amount for type 2 diabetes patients performing light work (23). We divided the patients into three groups using 25 kcal/kgIBW and 30 kcal/kgIBW as cutoff values and examined their relationships with survival duration. Since there is no precedent study about the cutoff value for the difference from TEE, we divided the patients into three groups using −300 kcal and 0 kcal as the cutoff values, considering the amount that can be supplemented with supplementary food. Furthermore, to evaluate the independence of the risk of less than 25 kcal/kgIBW and the benefit of calorie intake more than the Shimizu formula, we performed a Cox multivariate analysis including age, ΔALSFRS-R, ΔBW, presence or absence of dysphagia, the time from onset to calorie evaluation, % V.C., and gender as variables. All tests were deemed significant at *p* ≤ 0.05.

## Results

**Figure 1:**
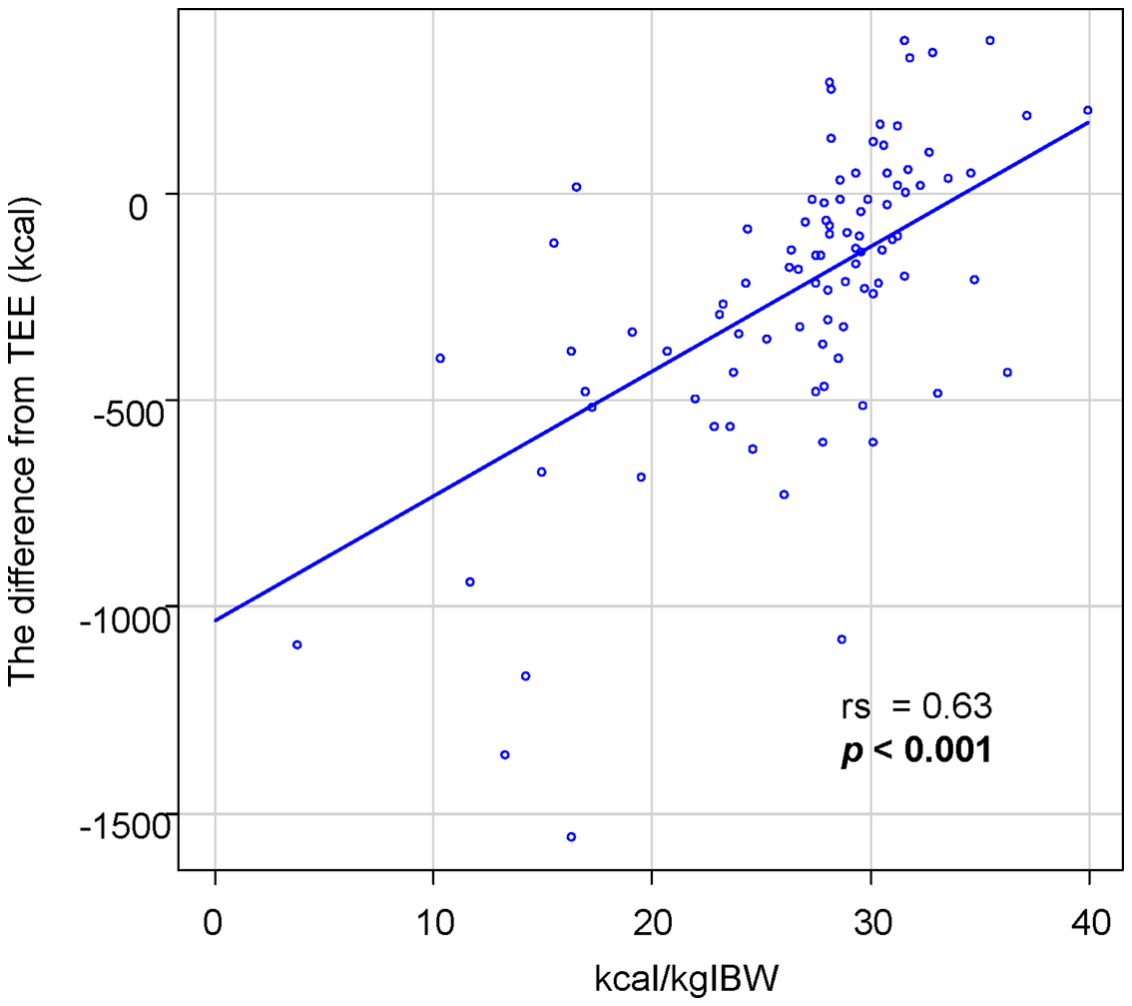
The association between oral intake per IBW and the difference from TEE. The oral intake per IBW is significantly correlated with the difference between oral intake and TEE.

Of the 105 patients initially admitted, one patient was excluded from the study due to inability to consume orally at the time. The demographic information is shown in Table 1. In 5 cases, the gastrostomy was performed within one week after admission. The median oral intake per IBW was 28.1 kcal/kgIBW. Among the participants, 29 patients consumed less than 25 kcal/kgIBW, 44 consumed equal to or more than 25 but less than 30 kcal/kgIBW, and 31 consumed equal to or more than 30 kcal/kgIBW. The intake was significantly higher in men at 28.8 kcal/kgIBW compared to women at 27.6 kcal/kgIBW (*p* = 0.047). For 94 patients whose required calorie intake was calculated using the Shimizu method, the median difference between oral intake and TEE was −172 kcal. Despite encouragement to increase caloric intake, only 24 of the 94 patients (25.5%) consumed equal to or more than their TEE. We investigated potential differences in the frequency of dysphagia and the factors encompassed by the Shimizu formula between two groups: those with oral intake equal to or exceeding the Total Energy Expenditure (TEE) and those with intake below TEE (Table 2). The group with an oral intake at or above TEE was notably older and exhibited a lower body weight. When stratified by oral intake per IBW, only 1 out of 25 patients (4.0%) consuming less than 25 kcal/kgIBW had this surplus, compared to 23 out of 69 (33.3%) in the group consuming equal to or more than 25 kcal/kgIBW, which was significantly higher (*p* = 0.003). There was a positive correlation between oral intake per IBW and the difference between TEE and oral intake (Figure 1, rs = 0.63, *p* < 0.001).

**Table 1:** Characteristics.

		*n*	
Age (years)		104	70 (61, 75)
Women			50/104 (48.1%)
ALSFRS-R		94	41 (37, 44)
ΔALSFRS-R since onset (/month)		94	0.56 (0.29, 1.00)
B.W. before the onset (kg)		103	59.0 (51.5, 68.0)
B.W. at the first visit (kg)		104	53.6 (46.9, 64.1)
ΔBW since onset to the first visit(kg/month)		103	0.33 (0.00, 0.74)
B.W. at the oral intake assessment (kg/m^2^)		104	54.0 (46.3, 64.1)
Duration since onset to the first visit (months)		104	10 (6, 16)
Duration since onset to the caloric assessment (months)		104	13 (8, 21)
Dysphagia			36/104 (34.6%)
El Escorial criteria	Possible		19/ 104 (18%)
	Probable		66/104 (64%)
	Definite		19/ 104 (18%)
PEG since this admission			64/104 (62%)
%VC		95	86.5 (74.0, 95.3)
**Metabolism**			
mREE (kcal)		83	1,280 (1,090, 1,452)
LSTM (kg)		81	34.8 (28.6, 40.6)
mREE/LSTM (kcal/kg)		81	37.8 (34.3, 41.3)
Body temperature (°C)		104	36.53 (36.38, 36.68)
HDL (mg/dL)		102	63 (50, 74)
LDL (mg/dL)		102	116 (96, 139)
LDL/HDL		102	1.91 (1.43, 2.3)
**Oral intake**			
Oral intake per IBW (kcal/kgIBW)		104	28.1 (24.2, 30.4)
Difference from TEE (kcal)		94	−172 (−399, −3.00)
The genesis of endpoint			59/104 (57%)

The data is presented as the median (interquartile range). Due to missing values, the “*n*” for each factor does not necessarily amount to the total of 104. ALSFRS-R, revised amyotrophic lateral functional rating scale; B.W., body weight; IBW, ideal body weight; LSTM, lean soft tissue mass; mREE, measured resting energy expenditure; PEG, percutaneous endoscopic gastrostomy. TEE, total energy expenditure; V.C., vital capacity.

**Table 2:** Differences in clinical characteristics based on whether dietary intake exceeded TEE.

	≥ TEE	< TEE	*p*
**Age (years)**	74 (69, 77)	67 (59, 74)	**0.006**
ALSFRS-R	40 (36, 43)	41 (38, 44)	0.24
**Body weight (kg)**	49.5 (45.5, 57.4)	56.55(47.7, 65.3)	**0.025**
Dysphagia	7/24 (29%)	24/70 (34%)	0.80
Hight (m)	1.61 (1.54, 1.66)	1.60 (1.54, 1.68)	0.50
Women	9/24 (38%)	35/70 (50%)	0.35

Bold values are significant differences.

Next, we studied the relationship between oral intake per IBW and metabolic factors, progression rate, and rate of weight loss (Table 3). Positive correlations were found with LDL (rs = 0.21, *p* = 0.039), LDL/HDL (rs = 0.29, *p* = 0.003), and %VC (rs = 0.27, *p* = 0.009). Negative correlations were observed with body temperature (rs = −0.28, *p* = 0.004), ΔALSFRS-R (rs = −0.39, *p* < 0.001), and ΔBW (rs = −0.34, *p* < 0.001). HDL (rs = −0.17, *p* = 0.080) and LSTM (rs = 0.23, *p* = 0.052) also seemed to be associated with oral intake per IBW, although insignificant. There was no significant correlation with mREE or mREE/LSTM. The difference from TEE did not show any significant correlations. Body temperature positively correlated with mREE/LSTM (rs = 0.25, *p* = 0.022).

**Table 3:** Correlation with oral intake.

	Per IBW		Difference from TEE	
	rs	*p*	rs	*p*
HDL	−0.17	0.080	< 0.001	0.998
**LDL**	**0.21**	**0.039**	0.04	0.672
**LDL/HDL**	**0.29**	**0.003**	0.06	0.56
mREE	0.17	0.134	−0.20	0.080
LSTM	0.22	0.052	−0.146	0.198
mREE/LSTM	−0.101	0.37	0.03	0.82
**Body temperature**	**−0.28**	**0.004**	−0.13	0.21
**%VC**	**0.27**	**0.009**	0.10	0.35
**ΔALSFRS-R**	**−0.39**	**<0.001**	−0.11	0.27
**ΔBW**	**−0.34**	**<0.001**	−0.07	0.48

Bold values are significant differences.

**Figure 2:**
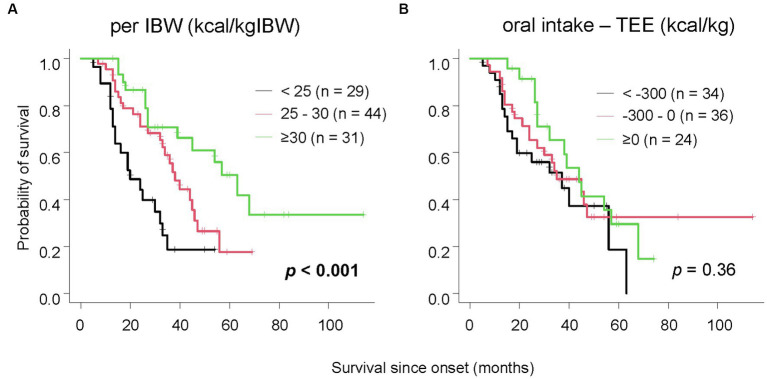
The relationship between survival time and oral intake. Patients with lower oral intake per IBW had significantly shorter survival **(A)**. When classified by the difference between oral intake and TEE, no significant difference in survival was observed **(B)**.

**Figure 3:**
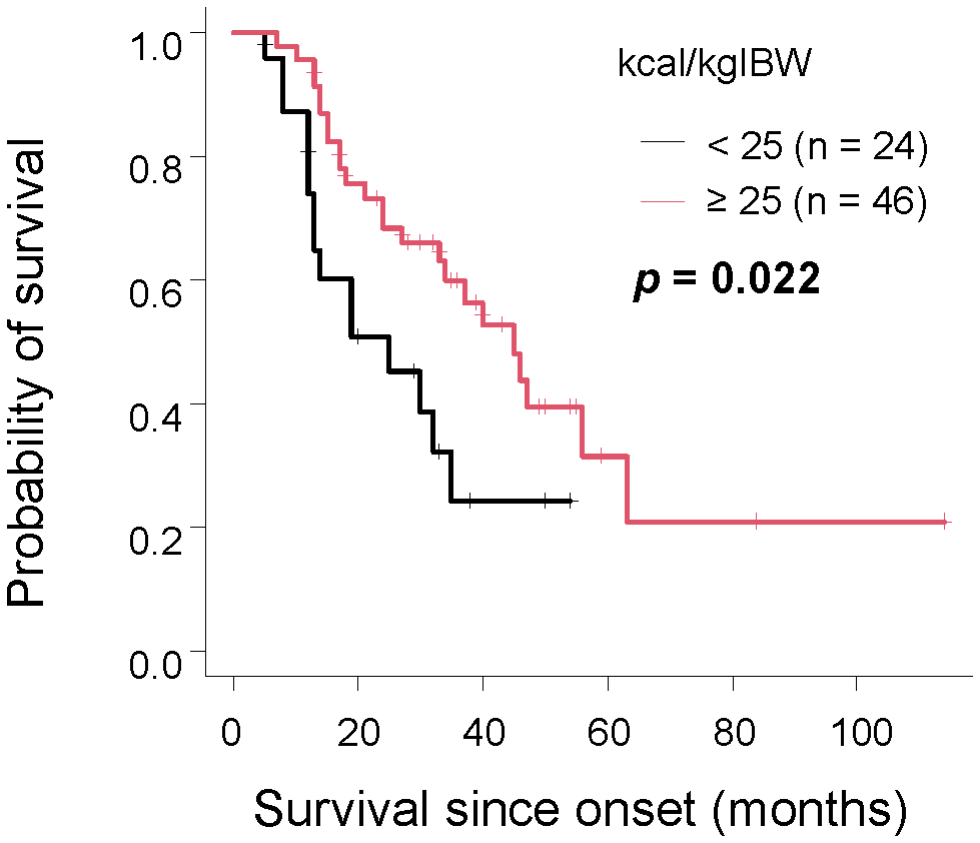
The relationship between survival time and oral intake per IBW in the group with unmet TEE In the group where TEE was not met, patients with an oral intake per IBW of less than 25 kcal/kgIBW had a significantly shorter survival compared to those with the same or more.

Furthermore, we investigated the relationship between survival duration and oral intake. The median survival time for patients consuming less than 25 kcal/kgIBW was 20 months, 38 months for those consuming 25-30 kcal/kgIBW, and 63 months for those consuming equal to or more than 30 kcal/kgIBW, showing a significant difference (Figure 2A, *p* < 0.001). Even after excluding 17 patients with extremely low dietary intake of less than 20 kcal/kgIBW, the group consuming less than 25 kcal/kgIBW had a significantly shorter median survival time as long as 19 months (Supplemental Figure, *p* < 0.001). There was no significant difference in survival duration between groups, stratified by the difference from TEE (Figure 2B, *p* = 0.36). Among the 70 patients who consumed less than their TEE, a significant difference in median survival was observed between those who consumed less than 25 kcal/kgIBW (25 months) and those who consumed equal to more (45 months) (Figure 3, *p* = 0.022). Among the 24 patients who consumed more than their TEE, only one whose TEE was about 820 kcal consumed less than 25 kcal/kgIBW. Multivariate Cox analysis showed that an oral intake of less than 25 kcal/kgIBW was an independent poor prognostic factor (Table 4, hazard ratio; H.R.: 3.45; *p* = 0.012). A tendency for a favorable prognosis was observed with consumption greater than TEE, though it wasn’t significant (Supplemental Table, H.R.: 0.51; *p* = 0.062).

**Table 4:** Multivariate Cox analysis.

Multivariate analysis	Adjusted H.R.	*p*
Age	1.02 (0.98–1.07)	0.27
ΔALSFRS-R	1.19 (0.77–1.85)	0.44
**ΔBW (/months)**	**2.03 (1.01–4.08)**	**0.047**
Dysphagia	0.99 (0.47–2.06)	0.97
**Oral intake < 25 kcal/kgIBW**	**3.45 (1.31–9.06)**	**0.012**
**Time since onset to the caloric assessment (months)**	**0.89 (0.85–0.93)**	**<0.001**
%VC	1.00 (0.98–1.03)	0.79
Women	0.55 (0.27–1.14)	0.106

*p*-values are based on Cox PH models. P.H., proportional hazards; H.R., hazard ratio (95% confidence interval); ALSFRS-R, revised amyotrophic lateral functional rating scale; B.W., body weight; IBW, ideal body weight; V.C., vital capacity.Bold values are significant differences.

**Figure 4:**
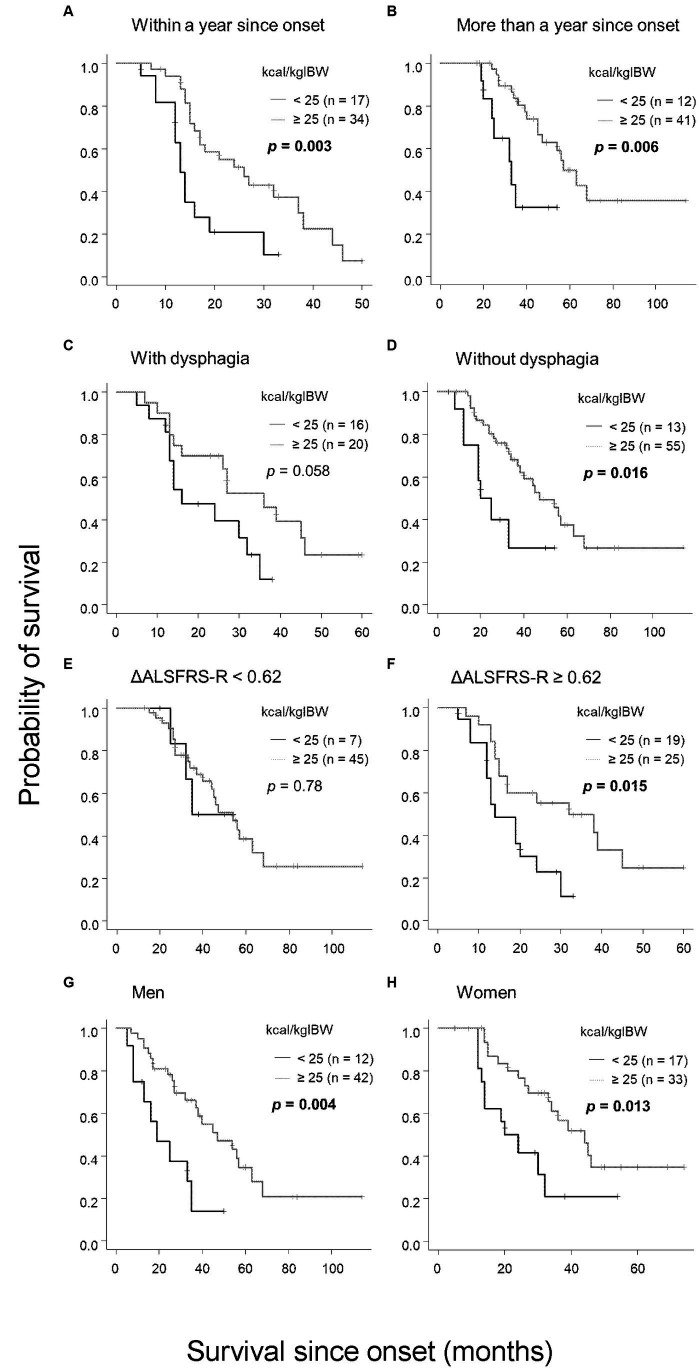
Subgroup analysis of the relationship between oral intake per IBW and survival. Patients with an oral intake per IBW of less than 25 kcal/kgIBW had significantly shorter survival compared with those with 25 kcal/kgIBW or more in the following categories: **(A)** within 1 year of onset, **(B)** more than 1 year from onset, **(D)** absence of dysphagia, **(F)** ΔALSFRS-R≧0.62, **(G)** male, and **(H)** female. In patients with dysphagia **(C)**, the trend was the same, although no significant difference was observed. In the group with ΔALSFRS-R < 0.62, no significant relationship between oral intake and survival was observed **(E)**.

Given the significant finding that patients with an oral intake of less than 25 kcal/kgIBW had shorter survival durations, we performed subgroup analyses considering the time from onset to calorie evaluation, presence or absence of dysphagia, progression rate differences, and gender (Figure 4). Based on prior research, we categorized the time from onset as less than or more than 1 year (24) and defined rapid progression as ΔALSFRS-R≧0.62 and slow progression as less (6). Groups with onset time equal or less than 1 year (*p* = 0.003), more than 1 year (*p* = 0.006), rapid progression (*p* = 0.006), men (*p* = 0.004), and women (*p* = 0.013), and without dysphagia (*p* = 0.016) all showed that patients with an oral intake of less than 25 kcal/kgIBW had significantly shorter survival durations than other groups. Similar tendencies, though not significant, were observed in patients with dysphagia (*p* = 0.058). No significant relationship was observed in the slow progression group (*p* = 0.78).

## Discussion

This study investigated the previously undetailed relationship between oral intake and prognosis in ALS. Patients with lower intake per IBW, particularly under 25 kcal/kgIBW, had notably shorter survival, consistent across disease duration from onset and gender. However, the difference derived from the Shimizu formula for TEE did not significantly affect survival.

The results of this study suggest that oral intake per IBW can be useful in predicting prognosis. Of course, in the group where dietary intake had already decreased at the time of first admission, this may be due to the inclusion of patients with rapid progression, severe dysphagia, or those who have progressed to the point where they are unable to eat. However, subgroup analysis showed that groups with low oral intake per IBW tended to have poor prognosis, even when stratified by the disease progression rate or presence or absence of dysphagia (Figure 4). Furthermore, in the Cox multivariate analysis, an oral intake of less than 25 kcal/kgIBW was an independent poor prognostic factor, regardless of age, progression rate, weight loss rate, presence of dysphagia, and % V.C. (Table 4). Even excluding patients with an extremely low dietary intake below 20 kcal/kgIBW, the correlation between oral intake per IBW and prognosis remained unchanged. This suggests that having a low oral intake per IBW itself is a significant issue related to disease progression in ALS patients. Considering the positive correlation of this index with LDL, LDL/HDL ratio, and LSTM, and a negative correlation with weight loss rate (Table 3), a decrease in oral intake per IBW may directly lead to malnutrition and affect prognosis.

On the other hand, the difference between TEE and oral intake showed no significant relationship with survival period and was not correlated with lipid profile, progression rate, or weight loss rate. In this cohort, although meals were provided to aim for a TEE, only 25.5% of the total could achieve oral intake above TEE. Patients who were younger and had higher body weights demonstrated a decreased likelihood of achieving TEE. Deciding whether to increase nutritional intake in these patients or use a gastrostomy to exceed the TEE is a significant clinical challenge. Patients with an oral intake of 25 kcal/kgIBW or more, even if below the TEE, had survival rates similar to average ALS patients, suggesting this intake level may be adequate. It should be noted that the Shimizu formula, using the double-label method to estimate energy expenditure for Japanese ALS patients, is highly accurate. Despite the advantages of high-calorie therapy (6, 25), the main objective is to match or surpass the TEE from the Shimizu formula, setting a baseline of 25 kcal/kgIBW for oral intake.

Larger energy intake to maintain energy balance is crucial, particularly in the light of the observed hypermetabolism and increased physical activity-energy expenditure in ALS patients compared to controls (26). Altered metabolism is also evident in the presymptomatic stage, demonstrated by studies indicating reduced lean body mass in presymptomatic ALS gene carriers (27). Moreover, BMI has shown a decline in the 5 years prior to diagnosis (28). These findings highlight the potential need for gastrostomy in individuals with inadequate caloric intake. Consistent with this, multiple observational studies have indicate that gastrostomy helps maintain weight in ALS patients (29) and prolongs survival (30–32); the American Neurological Association also endorses it for ALS patients (5). For patients with dysphagia, survival is longer with gastrostomy (33–36), and it is advised for those with over 10% weight loss (37) or BMI below 18.5 (2). However, it’s impractical to immediately use gastrostomy upon detecting dysphagia, and its efficacy may be diminished in instances of low BMI. The optimal timing remains undefined in both scenarios. Future multi-center studies should explore if initiating gastrostomy based on an IBW oral intake of 25 kcal/kgIBW indicator can extend survival.

This study also explored the uncharted link between oral intake and metabolism. While no direct correlation emerged between oral intake per IBW and mREE or mREE/LSTM (Table 3), a notable negative association was found between oral intake per IBW and body temperature. Intriguingly, body temperature exhibited a positive correlation with mREE/LSTM. To the best of our knowledge, the connection between body temperature and hypermetabolism has not been previously documented in the context of ALS. Our results suggest a potential link between body temperature and resting energy expenditure, shedding light on a novel aspect of ALS metabolism. Additionally, the negative correlation between oral intake and body temperature suggests an underlying link between oral intake and metabolism in ALS patients.

This study bears limitations. Firstly, this retrospective, single-center study was conducted solely on Japanese subjects. The Shimizu formula, which was developed based on data from Japanese patients—typically exhibiting lower body weights than Caucasians—necessitates careful interpretation of results with consideration for regional variations. Secondly, the assessment relied on a 7-day dietary intake visually evaluated by ward nurses during diagnostic hospital stays, which might not reflect typical home consumption. However, patients and their families were instructed on how to assess calorie intake at home to make the results of this study useful in regular healthcare. Thirdly, differing PEG timings precluded us from examining its effect on survival length. Fourthly, measuring body temperature in the axillary area can be affected by external conditions like room temperature. Fifthly, pre-morbid body weight may not be entirely accurate as patients might not remember their former weight precisely. Finally, we informed all the patients of the significance of caloric intake, and urged them to adhere; yet, the reactions and effectiveness might have differed among individuals, potentially influencing the results.

In conclusion, the study shows that ALS patients consuming less than 25 kcal/kgIBW have shorter survival spans, which may be associated with malnutrition. Notably, even if patients did not achieve the TEE as per the Shimizu formula, outcomes were favorable as long as oral intake matched or surpassed this benchmark. Proactive nutritional interventions, like gastrostomy, using the 25 kcal/kgIBW marker might aid in enhancing prognosis where reaching the full TEE is difficult.

## Data availability statement

The raw data supporting the conclusions of this article will be made available by the authors, without undue reservation.

## Ethics statement

The studies involving humans were approved by Shiga University of Medical Science Research Review Board. The studies were conducted in accordance with the local legislation and institutional requirements. The participants provided their written informed consent to participate in this study.

## Author contributions

RN: Conceptualization, Formal analysis, Project administration, Writing – original draft. MK: Data curation, Methodology, Supervision, Writing – review & editing. SK: Investigation, Supervision, Writing – review & editing. YT: Investigation, Supervision, Writing – review & editing. NO: Investigation, Supervision, Writing – review & editing. AK: Conceptualization, Methodology, Supervision, Writing – review & editing. IY: Methodology, Supervision, Writing – review & editing. SB: Conceptualization, Formal analysis, Methodology, Supervision, Writing – review & editing. TT: Investigation, Supervision, Writing – review & editing. MU: Data curation, Formal analysis, Funding acquisition, Investigation, Methodology, Supervision, Validation, Writing – original draft, Writing – review & editing.
