# Supplementary material for: Ideal body weight-based determination of minimum oral calories beneficial to function and survival in ALS
Source: Front Neurol. 2023 Nov 8;14:1286153. doi: 10.3389/fneur.2023.1286153 (PMC10663338; doi:10.3389/fneur.2023.1286153)
Supplement: Supplementary file 1 [file Data_Sheet_1.pdf]

Supplemental Fig.

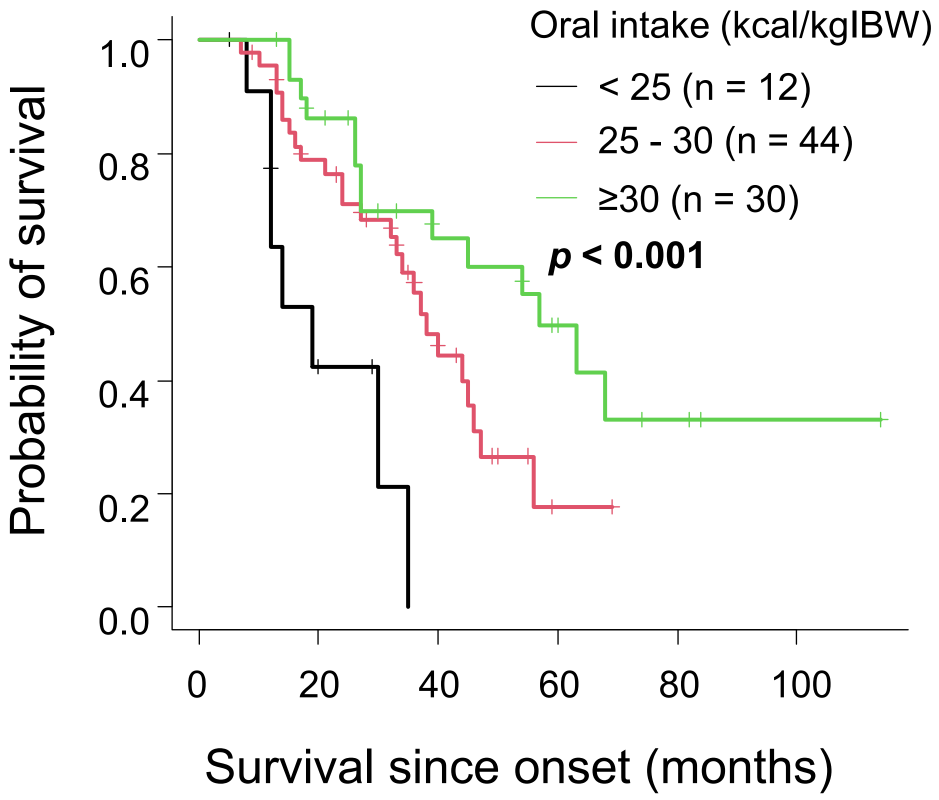

Supplemental Table

Multivariate analysis	Adjusted HR	*p*
Age	1.02 (0.99- 1.07)	0.21
Δ ALSFRS-R	1.34 (0.82- 2.20)	0.25
Δ BW(/months)	2.45 (1.21- 4.97)	0.013
Dysphagia	0.92 (0.44- 1.94)	0.82
Oral intake – TEE ≥ 0 (kcal/kgIBW)	0.51 (0.25- 1.03)	0.062
Time since onset to the caloric assesment (months)	0.90 (0.86- 0.95)	<0.001
%VC	1.00 (0.97-1.02)	0.77
Women	0.61 (0.31- 1.22)	0.163

P-values are based on Cox PH models. PH, proportional hazards; HR, hazard ratio (95% confidence interval); ALSFRS-R, Revised Amyotrophic Lateral Functional Rating Scale; BW, body weight; IBW, ideal body weight; VC, vital capacity.
